# Cost-effectiveness and affordability of anticancer treatment in Brazil

**DOI:** 10.3332/ecancer.2020.ed96

**Published:** 2020-01-20

**Authors:** Pedro Nazareth Aguiar

**Affiliations:** Américas Centro de Oncologia Integrado, Rua Martiniano de Carvalho, 741 Bela Vista, São Paulo, SP 01321-001, Brazil; Faculdade de Medicina do ABC, Av Príncipe de Gales, 821 Vila Principe de Gales, Santo André, SP 09060-650, Brazil

**Keywords:** cost-effectiveness, affordability, patient access, cancer treatment

## Abstract

The cost of anticancer treatments has increased in recent years. This is a threat to the sustainability of health systems. The number and relevance of pharmacoeconomic studies has increased, although their interpretation has become more complex. In a majority of cases, the benefit provided by new drugs is not enough to consider them cost-effective. In other cases, the treatment can be cost-effective, but the budget impact is unaffordable. Both cases deserve a deep discussion on how to make these treatments available to patients.

Cancer incidence is on the rise in higher and middle-income countries [1]. New anticancer treatments are being launched more frequently, with increased costs. In a recent study, Prasad et al showed that between 2010 and 2014, the median monthly cost of new anticancer drugs in the USA exceeded $10,000 [2]. In lower- and middle-income countries, a new drug is often unaffordable considering the purchasing power parity. [3]. In addition, new technologies lead to increased costs. [4]. Hence, the cost of anticancer treatments may exceed the household income, [2] threatening the sustainability of the healthcare system [5].

In addition to increased costs, another challenge is the assessment of treatment efficacy. Currently, pharmaceutical industries are developing complex study designs to accelerate drugs approval by regulatory agencies. Although many drugs are approved based on still preliminary data, oncologists agree that most of them promote improvement in patient outcomes [4]. Pharmacoeconomic studies assesses whether the magnitude of benefit given by a health technology is proportional to its increased cost [6].

In recent years, an increasing number of studies have been dedicated to pharmacoeconomics. In addition, international oncology conferences allow discussions regarding issues related to the pharmacoeconomics of new anticancer agents. There are several types of pharmacoeconomic studies, including cost-effectiveness, cost-utility, and cost-benefit studies [6].

In cost-effectiveness studies, outcomes are measured in units of time, while in cost-utility studies, outcomes are adjusted as a measure of the quality of life the treatment provides. [6]. In cost-benefit studies, outcomes are measured in monetary units. Therefore, it is challenge to conduct cost-benefit studies in practice [6]. In Oncology, it is important to consider potential adverse events of anticancer drugs as well as patients’ quality of life. Therefore, cost-utility studies are most commonly employed [7]. The endpoint of cost-utility studies is the Incremental Cost-Effectiveness Ratio (ICER), and it is defined by the amount of money required to gain one year of life plenty of quality [6].

It is important to consider the parameters used in a cost-effectiveness study [6]. The study must define its perspective (e.g., private insurance perspective or public health perspective) and clarify all costs (e.g., direct costs such as drugs acquisition costs or indirect costs such as inpatient costs due to adverse events) [6].

Another important consideration for cost-effectiveness is the accepted threshold. According to the World Health Organization, the threshold for cost-effectiveness is less than three times the gross domestic product (GDP) *per capita* [8, 9]. For a drug to be cost effective for the Brazilian per capita GDP, its ICER must not exceed $30,000. Considering the high cost of new anticancer drugs, it is unlikely that novel anticancer therapies are cost-effective in Brazil [10].

In contrast, treatments for common types of cancer, including breast cancer, may prove to be unaffordable for healthcare systems despite being cost-effective [8]. To incorporate a new technology, the Brazilian Public Healthcare System regulatory agency usually assesses the budget impact along with cost-effectiveness [11].

Consequently, experts and policy makers are discussing the technical and socioeconomic aspects associated with the incorporation of new drugs in the healthcare system [8].

The two possible solutions are as follows:

- Benchmark interventions: to consider cost-effective all innovative drugs with an ICER below the ICER of drugs previously incorporated
- League table interventions: to discuss and rank novel treatments according to associated ICERs and incorporate new drugs following a sequence of priority

It is important to highlight that the health technology assessment after the approval by the National Health Surveillance Agency is mandatory only to the Brazilian Public Healthcare System, which includes 75% of the population. In Brazil, the private health sector must make anticancer medications available to patients after the approval by the National Health Surveillance Agency.

Finally, we conclude that patients’ access to innovative anticancer drugs may be seriously compromised due to increased costs of each new compound. In many cases, the incorporation of a new health technologies must not depend solely on cost-effectiveness studies.

## Conflicts of interest

The author states that there were no conflicts of interest regarding this editorial. The author did not receive any honoraria or travel expenses from the Pharmaceutical Industry in the last year.

## Funding declaration

This editorial was made with author’s resources with no external funding.
